# Ecologically Appropriate Xenobiotics Induce Cytochrome P450s in *Apis mellifera*

**DOI:** 10.1371/journal.pone.0031051

**Published:** 2012-02-03

**Authors:** Reed M. Johnson, Wenfu Mao, Henry S. Pollock, Guodong Niu, Mary A. Schuler, May R. Berenbaum

**Affiliations:** 1 Department of Entomology, University of Illinois at Urbana-Champaign, Urbana, Illinois, United States of America; 2 Department of Cell and Developmental Biology, University of Illinois at Urbana-Champaign, Urbana, Illinois, United States of America; AgroParisTech, France

## Abstract

**Background:**

Honey bees are exposed to phytochemicals through the nectar, pollen and propolis consumed to sustain the colony. They may also encounter mycotoxins produced by *Aspergillus* fungi infesting pollen in beebread. Moreover, bees are exposed to agricultural pesticides, particularly in-hive acaricides used against the parasite *Varroa destructor*. They cope with these and other xenobiotics primarily through enzymatic detoxificative processes, but the regulation of detoxificative enzymes in honey bees remains largely unexplored.

**Methodology/Principal Findings:**

We used several approaches to ascertain effects of dietary toxins on bee susceptibility to synthetic and natural xenobiotics, including the acaricide tau-fluvalinate, the agricultural pesticide imidacloprid, and the naturally occurring mycotoxin aflatoxin. We administered potential inducers of cytochrome P450 enzymes, the principal biochemical system for Phase 1 detoxification in insects, to investigate how detoxification is regulated. The drug phenobarbital induces P450s in many insects, yet feeding bees with phenobarbital had no effect on the toxicity of tau-fluvalinate, a pesticide known to be detoxified by bee P450s. Similarly, no P450 induction, as measured by tau-fluvalinate tolerance, occurred in bees fed xanthotoxin, salicylic acid, or indole-3-carbinol, all of which induce P450s in other insects. Only quercetin, a common pollen and honey constituent, reduced tau-fluvalinate toxicity. In microarray comparisons no change in detoxificative gene expression was detected in phenobarbital-treated bees. However, northern blot analyses of guts of bees fed extracts of honey, pollen and propolis showed elevated expression of three CYP6AS P450 genes. Diet did not influence tau-fluvalinate or imidacloprid toxicity in bioassays; however, aflatoxin toxicity was higher in bees consuming sucrose or high-fructose corn syrup than in bees consuming honey.

**Conclusions/Significance:**

These results suggest that regulation of honey bee P450s is tuned to chemicals occurring naturally in the hive environment and that, in terms of toxicological capacity, a diet of sugar is not equivalent to a diet of honey.

## Introduction

*Apis mellifera*, the western honey bee, is the premier managed pollinator in the United States; the value of its contribution to agriculture in the form of providing pollination services to over 90 crop species exceeds $14 billion annually [1]. Stresses experienced by this species in the form of environmental toxins therefore have impacts across the agricultural spectrum. The ability of the honey bee to forage across the landscape leaves it vulnerable to exposure to a wide range of agricultural chemicals. For decades, pesticides used for control of crop pests and human disease vectors have caused honey bee mortality and morbidity [2], [3]. Despite labeling restrictions and a trend toward reduced use of pesticides in agriculture and forestry, pesticide applications continue to kill nontarget honey bee colonies [4]. Sublethal effects of pesticides exposure (e.g., neonicotinoids used for seed pretreatment) are suspected of causing reductions in hive viability [5]. Moreover, for the last two decades, the presence of *Varroa destructor*, a devastating parasitic mite that infests honey bee colonies, has led to additional xenobiotic stresses in the form of in-hive acaricide use, exposing bees to synthetic pesticides for the entire duration of their life cycle. The deliberate introduction of chemical pesticides to the hive environment has occurred largely without detailed knowledge of how honey bees process and thus tolerate these toxic compounds.

Like most other insects, honey bees rely in part on a suite of detoxification enzymes to metabolize naturally occurring xenobiotics and pesticides. Chief among these enzymes are the cytochrome P450 monooxygenases (P450) [6]. P450s play a role in the detoxification of phytochemicals [7] present in the nectar, honey and pollen that bees consume [8]–[10]. Additionally, the beehive, with its stores of pollen and beebread, provides a hospitable environment for fungi in the genus *Aspergillus*, which produce mycotoxins [11], [12] that are detoxified by P450s in the honey bee [13].

Synthetic pesticides are also metabolized by P450s in honey bees. Tau-fluvalinate, a pyrethroid acaricide that is used in-hive by beekeepers to control *Varroa* mites, is metabolized by P450s [14], [15], as are the pyrethroid lambda-cyhalothrin [16] and the organophosphate in-hive acaricide coumaphos [15], [17]. Indeed, tolerance of these acaricides is attributable in part to rapid P450-mediated detoxification by bees and is the reason these pesticides can be safely used in the hive by beekeepers for *Varroa* control. Many other organophosphate and pyrethroid pesticides are highly toxic to honey bees, although the toxicity varies according to the specific pesticide [18].

The apparent sensitivity of honey bees to some pesticides became the focus of discussion following the sequencing of the honey bee genome [19]. P450s are central to tolerance and evolved resistance to pesticides in many pest insects [20] but the *A. mellifera* genome encodes only 46 P450s, far fewer than most other insect genomes. Moreover, the carboxylesterase and the glutathione-S-transferase gene families, the other major detoxification genes in aerobic organisms, are similarly reduced in size [21]. It has been suggested that this reduced diversity of detoxification enzymes may contribute to the sensitivity of honey bees to certain pesticides [21] (but see [18]). Along with insights into honey bee biology, sequencing of the honey bee genome has also provided a wealth of new tools for investigating honey bee regulation of xenobiotic detoxification, a critical yet hitherto unexplored dimension of how this pollinator copes with a wide array of phytochemicals in its diet as well as synthetic pesticides and other xenobiotics. The paucity of genes in families associated with detoxification in combination with the existence of behavioral mechanisms of reducing toxin intake (e.g., [22]) suggests that regulation of these genes may differ in honey bees in comparison to nonsocial species and to herbivores that feed on chemically defended foliage.

Induction, the phenomenon whereby the production of a detoxification enzyme increases in response to toxin exposure [23], is thought to be widely associated with induced transcription of detoxification genes because it minimizes resource investment in superfluous metabolic capability and protects organisms from the oxidative damage that can accompany P450 activity [24]. Because P450 enzymes are frequently inducible by their substrates, induction has served as a useful tool in identifying specific P450s associated with pesticide tolerance and xenobiotic response [25]. To date, only one study has demonstrated induction of P450 activity in bees. Benzo(α)pyrene monooxidase activity in honey bee guts was induced by exposure to benzo(α)pyrene itself and by the in-hive acaricides tau-fluvalinate and cymiazole hydrochloride [26].

Phenobarbital, a synthetic barbiturate drug, is a potential inducer of P450 activity in honey bees in that it induces P450s in a wide range of organisms [27], [28]. In insects, phenobarbital induction increases enzymatic P450 activity in Diptera [29]–[32], Lepidoptera [33]–[37], and Blattodea [38]. Induction of P450 enzymatic activity has been measured either *in vitro* using pesticide metabolism assays, or *in vivo*, using pesticide toxicity as an indicator of detoxificative P450 activity [39]–[41]. Although phenobarbital is a reliable inducer of P450 activity in many insects, only a single study using phenobarbital has been performed in Hymenoptera; phenobarbital feeding had no effect on the toxicity or metabolism of carbaryl in alfalfa leafcutter bees (*Megachile rotundata*) [42].

In addition to inducing enzymatic activity, phenobarbital also induces transcription of P450 genes in many insects, with studies demonstrating elevated transcription of CYP6, CYP9 and CYP4 family P450s in both Lepidoptera [43]–[46] and Diptera [47]–[52]. Microarray studies with *Drosophila melanogaster* demonstrated induced expression of as many as 29 P450 genes following phenobarbital treatment [53]–[56]. To date, no P450 induction studies based on gene expression have been reported in honey bees or other hymenopterans treated with phenobarbital.

Natural phytochemicals that honey bees encounter in nectar, pollen and propolis may also serve as inducers of P450-mediated detoxification. Indeed, honey is known to be an effective P450 inducer in humans; elevated P450 enzyme activity was observed in humans after eating honey [57], although the specific components responsible for induction were not identified. Flavonoids, compounds important to plant resistance to insect herbivory [58], that are present in both pollen [8], [9], and honey [10], may induce P450s in bees. Lepidopteran larvae that consumed quercetin, a common flavonoid in foliage as well as honey and pollen, experienced increased P450 gene expression [45] as well as elevated P450 enzymatic activity against model substrates [59], [60]. Propolis, a resinous material collected by honey bees for use as a structural sealant and as an antibiotic [61], is rich in flavonoids and phenolic compounds and induces P450s involved in mycotoxin detoxification in this species [13].

Several classes of phytochemicals, which may not be present in nectar, pollen and propolis, act as inducers of P450-mediated metabolism in foliage-feeding insect herbivores. Xanthotoxin, a furanocoumarin produced by plants in the families Apiaceae and Rutaceae, is an effective inducer of xenobiotic-metabolizing P450s in several species of lepidopterans [45], [46], [62], [63] as are indole-3-carbinol [41], [45], [60], a derivative of the toxic glucosinolates produced by plants in the Brassicaceae, and salicylic acid [64], a ubiquitous phytohormone active in initiating plant defensive response to herbivory.

We examined the phenomenon of P450 induction in honey bees using two different approaches after the administration of chemicals that induce P450s in other organisms—by testing for functional evidence of induction by assaying tolerance of toxic compounds, and by measuring changes in P450 transcript abundance in response to candidate inducers. In the toxicity assays, we examined adult workers for the *in vivo* effects of putative inducers on the LD_50_ of pesticides known to interact with P450s. The toxicity of two pyrethroid pesticides detoxified by P450s in bees, tau-fluvalinate [14] and lambda-cyhalothrin [16], and two pesticides bioactivated by P450 activity in honey bees, imidacloprid [65] and aldrin [66], was assessed using this approach.

To determine whether as-yet unidentified phytochemicals in honey function as inducers, an additional set of toxicity bioassays was conducted. High-fructose corn syrup (HFCS) and sucrose syrup are commonly used in commercial apiculture [67]. Neither supplemental carbohydrate source contains the suite of plant secondary compounds that are present in nectar and honey and that may be important in P450 regulation. Susceptibility of adult workers to tau-fluvalinate and imidacloprid was compared on diets of honey, sucrose, and HFCS. In addition, longevity of adult worker bees in the presence of the naturally occurring mycotoxin aflatoxin B1, known to be metabolized by P450s [13], was compared on diets of honey, sucrose, and HFCS. We selected aflatoxin B1 for longevity assays as a toxin naturally present in the honey bee's environment to contrast with synthetic pesticides.

To evaluate effects of phenobarbital on P450 transcription, we analyzed bees exposed to phenobarbital using specialty honey bee microarrays [68]. To identify potential natural sources of P450 inducers, we also conducted northern blot analysis on expression levels of the CYP6AS subfamily of P450 genes in bees consuming extracts of honey, pollen and propolis. Mao et al. [7] demonstrated using heterologous expression that one P450, CYP6AS3, contributes to metabolizing quercetin, a flavonol that occurs widely in plant nectars, pollen and honey. The bee-specific expansion of a group of CYP6AS P450s in the honey bee genome, in contrast with the genome of the parasitoid wasp *Nasonia vitripennis* [69], suggests that other members of this clade of CYP6AS P450s may be involved in metabolism of diet constituents unique to the honey bee, including those found in honey, pollen and propolis.

## Results

### Functional P450 induction measured using LD_50_ bioassays

Of all of the potential inducers assayed, only one, quercetin, significantly decreased the toxicity of tau-fluvalinate to bees (Table 1). Pretreatment with indole-3-carbinol or salicylic acid did not alter the toxicity of tau-fluvalinate, while xanthotoxin and phenobarbital pretreatment actually increased the toxicity of tau-fluvalinate to bees. Phenobarbital feeding also increased the toxicity of lambda-cyhalothrin, although to a lesser degree than tau-fluvalinate. Toxicity of aldrin and its P450-bioactivated metabolite dieldrin increased similarly in bees fed phenobarbital.

**Table 1:** Toxicity of pesticides to *Apis mellifera* in the presence and absence of P450 inducers.

treatment	N	LD_50_ (95% CI) ng/bee	slope±SE	intercept±SE	X^2^	df
**tau-fluvalinate**	574	8050 (7210–8990)	2.54±0.21	−9.94±0.81	3.4	5
+ phenobarbital	661	190 (121–311)	1.46±0.12	−3.33±0.26	26	6
+ xanthotoxin	488	35.1 (0–126)	0.34±0.09	−0.52±0.23	8.4	6
+ quercetin	206	11400 (9740–13860)	2.98±0.40	−12.1±1.59	2.4	3
+ salicylic acid	260	4450 (2180–8560)	1.56±0.33	−5.68±1.33	14	4
+ indole-3-carbinol	84	8340 (5920–10930)	2.53±0.67	−9.93±2.66	1.5	2
**lambda-cyhalothrin**	75	47.5 (34.3–67.5)	2.46±0.57	−4.13±0.96	0.2	2
+ phenobarbital	238	16.9 (4.7–25.3)	2.95±0.39	−3.63±0.57	8.4	3
**aldrin**	911	60.5 (52.7–71.0)	5.64±0.35	−10.1±0.61	35	5
+ phenobarbital	467	38.5 (31.0–47.1)	3.91±0.36	−6.20±0.59	22	6
**dieldrin**	495	37.2 (31.9–46.5)	5.57±0.56	−8.75±0.85	22	5
+ phenobarbital	528	20.7 (14.7–25.8)	3.46±0.30	−4.56±0.43	21	5

Toxicity bioassays for the pyrethroid pesticides tau-fluvalinate and lambda-cyhalothrin, the organochlorine aldrin, and its bioactivated P450 metabolite dieldrin, using 3-day-old bees fed sucrose “bee candy” or candy with phenobarbital (5 mg/g candy), xanthotoxin (1 mg/g), quercetin (10 mg/g), salicylic acid (2.5 mg/g) or indole-3-carbinol (1 mg/g) added. N = total number of bees included in bioassay, LD_50_ = Lethal Dose 50%, as calculated by probit model, 95%CI = 95% confidence interval for the LD_50_ (treatments with non-overlapping 95% confidence intervals are considered significantly different) , slope = slope of the log-probit line, intercept = intercept of the log-probit line, SE = standard error, chi square = statistical test for the probit model, if significant then correction for heterogeneity using Fieller's method was applied, df = degrees of freedom for the chi square test.

In assays conducted to determine the ability of different diets (sucrose, HFCS, or honey) to induce P450s, one-way analysis of variance revealed no significant differences in tau-fluvalinate or imidacloprid toxicity to bees based on diet. Thus, there are apparently no constituents of honey that induce P450s that either detoxify or bioactivate these two pesticides.

### Functional P450 induction measured using longevity bioassays

A Kaplan-Meier Survival Analysis was conducted to examine differential sensitivity to aflatoxin based on diet. The median time for survival was calculated for each treatment independently. No significant differences in median survival time were found among bees consuming control-treated sucrose, HFCS, or honey (Table 2). However, in the presences of aflatoxin B1, bees maintained on a diet containing honey had a significantly longer median survival time (55.0 h) than bees fed diets containing HFCS (47.3 h) or sucrose (40.9 h) (Wilcoxon, p = 0.001). These findings suggest that honey contains one or more constituents that allow bees to better tolerate aflatoxin exposure, possibly through induction of P450s capable of detoxifying this mycotoxin.

**Table 2:** Median survival times for *Apis mellifera* fed various diets with and without aflatoxin B1 (AB1).

treatment	median survival (h)	std. error
sucrose	69.3	1.4
sucrose + DMSO	67.9	1.5
honey + DMSO	76.5	0.8
HFCS + DMSO	75.9	0.9
sucrose + AB1	40.9	1.1
honey + AB1	55.0	0.9
HFCS +AB1	47.3	0.6

Aflatoxin B1 was applied at 20 µg/g candy in 0.1% dimethyl sulfoxide (DMSO). A DMSO control was applied to diets of pure sucrose “bee candy”, or candy made from equal parts honey and sucrose or high fructose corn syrup (HFCS) and sucrose.

### Transcriptional P450 induction from phenobarbital measured using microarrays

Only a single gene, tetraspanin 16, was differentially expressed (p≤0.05, FDR) in response to phenobarbital; tetraspanin 16 showed a 1.4-fold induction in phenobarbital-treated bees. No P450s were differentially expressed, nor were any genes in other gene families associated with detoxification.

### Transcriptional P450 induction measured by northern blot

**Figure 1:**
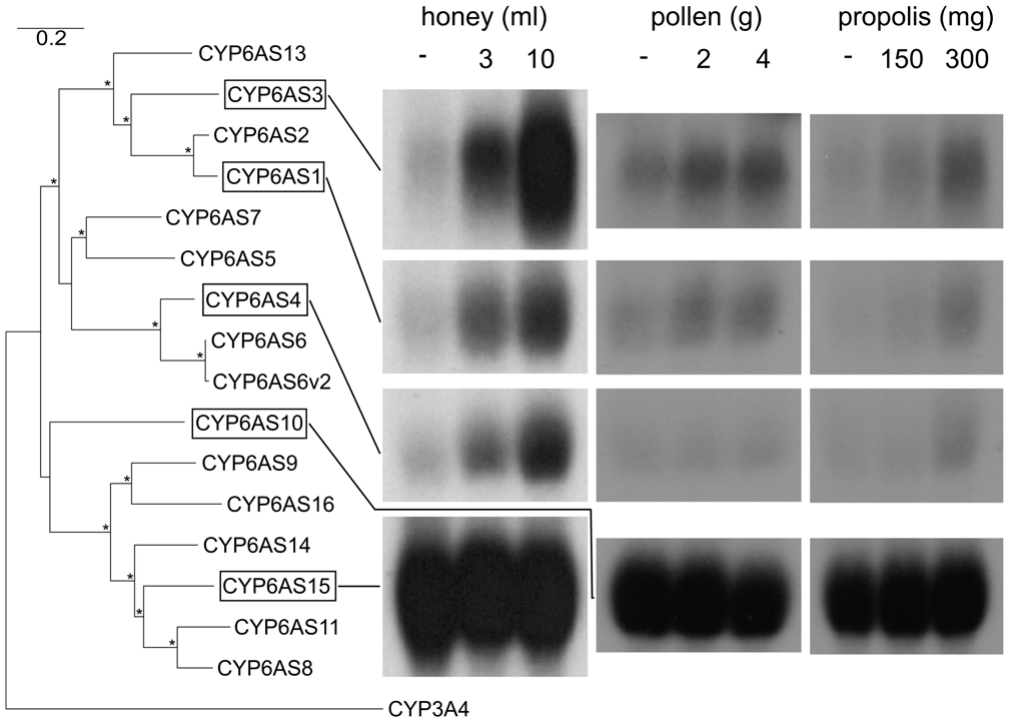
*Apis mellifera* CYP6AS family P450s and their gene expression following feeding on honey, pollen or propolis extract. Expression of selected P450 genes, as measured by northern blot, in guts of bees fed five g candy containing only sucrose or sucrose plus extract from the given quantity of honey, pollen or propolis. The neighbor-joining tree is rooted with *Homo sapiens* CYP3A4 and was created using CLUSTALW [88] alignment and PHYLIP [89] with 1000 bootstrap replicates. Branches with greater than 50% bootstrap support are indicated with an asterisk. Branch length in the final tree was corrected for multiple substitutions with TREE-PUZZLE [90].

Feeding on honey extract brought about substantial induction of CYP6AS3 and moderate induction of CYP6AS1 and CYP6AS4, in a dose-dependent manner (Figure 1). Expression of these P450 genes in the absence of honey extract was low. CYP6AS10 and CYP6AS15 were expressed at consistently high levels and their expression was not responsive to honey extract ingestion.

Pollen and propolis extract also caused slight induction of CYP6AS1 and CYP6AS3. Only propolis induced CYP6AS4. The induction brought about by low and high doses of pollen extract appeared the same, while only the highest dose of propolis extract resulted in induction of these genes. CYP6AS10 and CYP6AS15 were expressed at high levels and were not responsive to ingestion of either pollen or propolis extract.

### Visible effects of honey extract on the gut

**Figure 2:**
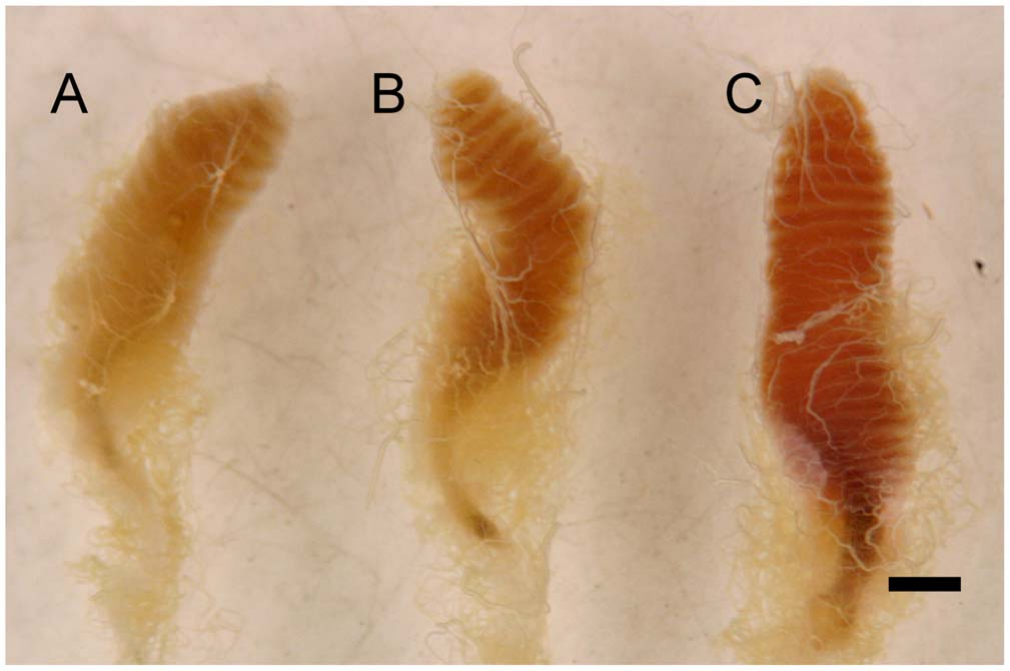
Dissected midguts of *Apis mellifera* fed sucrose “bee candy” or candy with honey extract. Midguts of bees fed on (a) plain sucrose candy were narrower than midguts of bees fed candy fortified with (b) a low dose (extract of 3 ml honey in 5 g sucrose candy) or (c) high dose of honey extract (10 ml honey). Scale bar = 1 mm.

Dissection of guts for extraction of RNA for microarray analysis suggested that the nature of the diet consumed by bees affects the morphology of the guts. Midguts of sucrose-fed bees, compared to those consuming honey extract, appeared fragile, flaccid and generally smaller. In order to quantify this apparent difference, a separate bioassay was conducted for the express purpose of quantifying morphological attributes of guts of bees fed different diets. Midguts of bees fed honey extract measured at their broadest point had a statistically greater diameter than did guts of bees fed plain “bee candy” (Figure 2; ANOVA, p<0.01, N = 41). Midguts of bees fed unaugmented candy (1.63 +/− 0.14 (SD) mm) were smaller in diameter than midguts from bees fed candy with a high dose of honey extract (1.82 +/− 0.17 mm; Tukey's HSD, p<0.01), while midguts of bees fed a low dose of honey extract were intermediate in width (1.75 +/− 0.15 mm).

## Discussion

In contrast with numerous studies finding phenobarbital induction of P450s in other insects, no P450s or any other detoxification genes showed a change in expression after phenobarbital feeding. Moreover, none of the pesticide toxicity bioassays showed evidence of P450 induction after phenobarbital exposure. Rather, phenobarbital treatment elevated the toxicity of all pesticides, suggesting that pesticides compete with phenobarbital for P450-mediated detoxification [70], [71]. Although phenobarbital has failed to induce P450-mediated detoxification in other insects (e.g., xanthotoxin detoxification in *Papilio polyxenes* [72]), phenobarbital enhancement of pesticide toxicity has not been previously reported.

Manipulation of honey bee diet yielded a dichotomy of responses in tolerance and toxicity, consistent with differential ability of dietary components to induce P450-mediated detoxification. Non-honey diets significantly decreased the ability of honey bees to tolerate the natural toxin aflatoxin B1 yet had no measurable effect on toxicity of the synthetic toxins tau-fluvalinate and imidacloprid. Although the number of substrates assayed is limited, this finding is consistent with the suggestion that regulation of honey bee P450s is more specialized than has been found in other insects. Such specialization may reflect the fact that honey bees for the most part consume only foods that they have processed—honey, beebread, or royal jelly. When a bee encounters a novel xenobiotic, such as the in-hive acaricides or imidacloprid, with which it has not coevolved, these synthetic compounds may not activate the same molecular pathways as are activated by naturally occurring xenobiotics in hive products.

Although honey bees tolerate a variety of synthetic pesticides [14], [17], [18], many others are extremely toxic to honey bees, including other pyrethroids similar to tau-fluvalinate [14]. Thus, metabolism of pesticides by honey bee P450s is most likely an incidental convergence of molecular structure and not an indicator of molecular evolution in response to selection. That aflatoxin B1 is detoxified by honey bee P450s [13] and not bioactivated, as it is by P450s in many other organisms, also is consistent with a coevolutionary history of adaptation.

The presence of substances in honey that induce or upregulate detoxificative P450s in honey bees raises the possibility that the longstanding practice of feeding bees sucrose or HFCS [73] may have unintended adverse impacts. beyond those already documented. Fructose in HFCS can be converted into a toxic by-product, 5-hydroxymethylfurfural, which can cause dysentery-like symptoms and mortality [74]. This study suggests that the survival of honey bees fed on HFCS and sucrose may be compromised when bees are also exposed to the fungal toxin aflatoxin B1, possibly because of reduced P450 activity and a resulting decreased capacity to tolerate aflatoxin. Because bees do not seem to be capable of inducing P450s in response to ecologically inappropriate xenobiotics, prophylactic induction of P450s through consumption of pollen and honey flavonoids may enhance bee survival.

Our findings are also consistent with the interpretation that honey bees, possibly due to the reduction in P450 genomic inventory, may rely on a small number of enzymes to detoxify both natural and synthetic xenobiotics. Because both aflatoxin [13] and acaricides such as tau-fluvalinate [14] are metabolized by P450s, there is potential for synergism between natural and synthetic xenobiotics, given that such synergism has been demonstrated between pyrethroid and organophosphate acaricides [17] and between pyrethroid insecticides and fungicides [75]. Recent dramatic declines in honey bee abundance associated with a suite of unusual attributes, collectively characterized as Colony Collapse Disorder [76], have led to speculation that pesticide exposures may be causing or contributing to bee losses [77]. Conducting an extensive survey across 23 states and a Canadian province over the 2007–2008 season, Mullin et al. [77] found “unprecedented levels of miticides and agricultural pesticides” in colonies. Residues of 118 different pesticides were recovered, with an average 6.5 pesticide detections per sample across wax, pollen, beebread, adult bees, and brood. Multiple exposures were typical; over 90% of the 749 samples analyzed contained at least two pesticides. These authors conclude their report with the statement that “the high frequency of multiple pesticides in bee collected pollen and wax indicates that pesticide interactions need thorough investigation before their roles in decreasing bee health can be either supported or refuted.” These levels of exposure, in the context of our findings that the ability of the honey bee to upregulate P450 detoxification genes in response to toxic exposure may be constrained and dependent in part on diet constituents, suggest that understanding precisely how honey bees process toxins, either individually or in combination, is a pressing necessity for maintaining the vitality of the U.S. apicultural enterprise.

## Methods

### Chemicals

Phenobarbital, xanthotoxin, quercetin, indole-3-carbinol and salicylic acid (Sigma, St. Louis, MO) were incorporated into confectioners sugar using a mortar and pestle, which was then used to make bee “candy.” A variety of doses were initially tested and a sublethal dose for each compound was chosen for the bioassays. Technical grade tau-fluvalinate (95%; Chem Service, West Chester, PA), aldrin and dieldrin were dissolved in chromatography-grade acetone for LD_50_ determination.

Tau-fluvalinate (95%) and imidacloprid (99.5%) were purchased from Chem Service (West Chester, PA). Aflatoxin B1 was purchased from Sigma Chemical Co. (St. Louis, MO). Tau-fluvalinate and imidacloprid stocks were dissolved in acetone purchased from Fisher Scientific (Pittsburgh, PA). Aflatoxin B1 stocks were dissolved in analytical grade dimethyl sulfoxide (DMSO) purchased from Fisher Scientific (Pitttsburgh, PA).

### Honey, pollen and propolis extract

Methanolic extracts were made from hive products collected from University of Illinois apiaries in 2007–2008. Honey, predominantly from soy and wildflower sources, was collected from University of Illinois apiaries in 2007. First, the honey was dissolved in distilled water to make a 10% solution. Diluted honey was filtered through a paper filter (Whatman, Kent, England) to remove particulate matter and then processed through a C_18_ silica column under vacuum. The column was washed with 5% methanol in water and then eluted with pure HPLC-grade methanol. Methanol was removed using a rotary evaporator (Büchi-Brinkmann, Flawil, Switzerland) and the remainder was resuspended in 1 ml methanol for each 20 ml of honey in the original solution. Ground pollen (Betterbee, Greenwich, NY) extract was made by extracting 10 g pollen in 100 ml of 90% methanol in water for 1 h at 25°C and then centrifuging and removing the liquid. This procedure was repeated three times. Raw pollen extract was then processed over a C_18_ silica column as was honey, with a final concentration of 2 g pollen for each 1 ml methanol. Propolis was scraped from frames and boxes of University of Illinois colonies located in a forested area in summer 2008. Propolis extract was made by freezing 3 g propolis with liquid nitrogen and grinding with a mortar and pestle. Propolis powder was dissolved in 30 ml methanol and heated just to boiling.

### Incorporation of xenobiotics into diet

Chemicals or extracts were administered in “bee candy” made from equal parts powdered sugar and heavy sucrose syrup with a ratio of 2∶1 sucrose to water (w/w). Sucrose (granulated table sugar) was processed in a blender to make starch-free powdered sugar. Approximately 5 g fresh liquid candy was poured into 2 oz (56 ml) plastic cups (Solo, Urbana, IL) and the candy was allowed to harden for at least 30 min before feeding to bees. Any candy not used immediately was stored at 4°C.

Phenobarbital, xanthotoxin, quercetin, salicylic acid, and indole-3-carbinol were incorporated into the powdered sugar component using a mortar and pestle. Final concentrations of treated candy fed to bees were the maximum concentration that did not cause increased mortality over control after 3 days and were as follow: 5 mg/g phenobarbital, 1 mg/g xanthotoxin, 10 mg/g quercetin, 2.5 mg/g salicylic acid, and 1 mg/g indole-3-carbinol.

Methanolic honey, pollen and propolis extracts were applied to powdered sugar, as was pure methanol as a control, and the solvent was allowed to evaporate overnight, prior to addition of heavy sucrose syrup. Bees were fed either a high or low dose of honey, pollen, or propolis extract, containing the extract of 3 or 10 ml honey, 2 or 4 g pollen, or 150 or 300 mg propolis per gram of candy.

Five microliters of aflatoxin B1 (20 µg/µl), or a DMSO control, was incorporated directly into the wet candy after addition of heavy sucrose syrup. Honey and HFCS were administered in the form of candy as well by using honey (University of Illinois apiaries) or high-fructose corn syrup (55% fructose, Archer Daniels Midland) in place of heavy sucrose syrup.

### Insects

Frames of late-stage sealed worker brood were taken from healthy colonies in the University of Illinois apiaries near Urbana, IL in July-August 2006, August-September 2008, and September-October 2009 and placed in a dark humid (∼80% RH) incubator maintained at 32–34°C. Newly eclosed adults were brushed from the frames at 24 h intervals and placed in screen-topped wooden boxes (330 cm^3^) in groups of 150–250. Newly emerged bees were immediately fed treated or control candy and maintained in the incubator for 3 days.

### LD_50_ determination

Full LD_50_ trials (Table 1) included an acetone control and doses causing 0% and 100% mortality, as well as at least four doses causing >0% and <100% mortality. Three- to four-day-old bees were anesthetized with CO_2_ in groups of 20 and 1 µl of tau-fluvalinate, lambda-cyhalothrin, aldrin, or dieldrin dissolved in acetone was applied to the thorax of each bee with a microliter syringe fitted in a Hamilton PB-600 repeating dispenser (Reno, Nevada).

Bees were also fed sucrose, HFCS, or honey candy and treated topically with three doses of tau-fluvalinate [14] (3, 5 and 10 µg) or imidacloprid [78] (0.005, 0.01 and 0.03 µg) ranging between the LD_25_ and the LD_50_. All trials included an acetone control, and no mortality was observed in any control bees.

Following treatment, bees were placed in wax-coated paper cups (177 cm^3^; Sweetheart, Owings Mills, MD) that were covered with cotton cheesecloth secured by two rubber bands. Sucrose water (1∶1 sucrose and water) was provided in a punctured 1.5 ml plastic tube. Bees were maintained in a dark 32–34°C incubator until mortality was assessed 24 h after treatment. Bees incapable of righting themselves inside the cup were scored as dead.

The R statistical package [79] with MASS libraries [80] was used to perform log-probit analyses of mortality data represented in Table 1. Fieller's method was used for calculation of LD_50_ values and 95% confidence intervals, with correction for heterogeneity where appropriate [81]. Non-overlapping 95% confidence intervals at the LD_50_ level were considered significantly different. SPSS 17.0 was used for bioassay analyses related to the effects of HFCS, sucrose and honey diets (SPSS Inc., Chicago, IL). One-way analysis of variance (ANOVA) was used to determine whether susceptibility to tau-fluvalinate and imidacloprid changes when bees are fed HFCS, sucrose or honey candy.

### Longevity assays

To test the effect of diet on longevity in the presence of aflatoxin, newly emerged bees were transferred in groups of 20 to wax-coated paper cups and fed one of seven treatments: sucrose candy, HFCS candy or honey candy, with the addition of either 0.1% DMSO as a control or 20 µg/g aflatoxin B1. A group fed on pure sucrose candy was also included. After treatment, bees were placed in an incubator and monitored in 6 h intervals for 72 h.

A Kaplan-Meier Survival Analysis was conducted to examine differential survivorship in the presence of aflatoxin based on diet. The median time for survival was calculated for each treatment independently (Table 2).

### Microarray construction

Experiments were designed to meet Minimum Information About a Microarray Experiment (MIAME) standards and all microarray data obtained in these studies were deposited in NCBI-GEO (http://www.ncbi.nlm.nih.gov/projects/geo Accession Number GSE34029). A custom microarray [68] was constructed by The Functional Genomics Unit of the W. M. Keck Center. Included on the array were probes specific for 45 P450 genes, 10 carboxylesterase genes and 7 glutathione-S-transferase genes along with 206 chemosensory-related genes and 17 tetraspanins, as well as houskeeping genes and controls, for 313 genes in total, using the *A. mellifera* assembly 2 as the basis for probe design [19].

### Microarray RNA isolation

Frames of brood from five different colonies were collected and newly emerging adults were fed phenobarbital (2.5 µg/g candy) or plain candy for three days as described. Total RNA was then isolated from 10 whole honey bee workers by first grinding in liquid nitrogen using a mortar and pestle and then extracting RNA with Trizol (Invitrogen, Carlsbad, CA), following the manufacturer's instructions. RNA concentration was quantified on a spectrophotometer and visually assessed on agarose gels.

### cDNA synthesis for microarrays

Fifteen µg of RNA from each treatment was reverse-transcribed into cDNA overnight at 46°C using SuperscriptIII (Invitrogen, Carlsbad, CA) with an oligo-dT16 primer and amino-allyl dNTP. cDNA was purified over a Qiaquick PCR purification kit column (Qiagen, Valencia, CA), substituting a phosphate wash for the provided kit buffers and then dried in a SpeedVac. The cDNA was labeled with either Cy-3 or Cy-5 mono-reactive dyes (GE Healthcare, Piscataway, NJ) in sodium carbonate buffer for 1 h in complete darkness and then purified over a PCR purification kit column; the cDNA concentration and labeling efficiency were quantified by spectrophotometer.

### Microarray hybridization

Spotted oligonucleotides were rehydrated by passing microarray slides through steam and then cross-linked by UV light exposure. Slides were then vigorously washed in 0.2% SDS, placed in prehybridization buffer for 1 h at 42°C, washed in ultrapure water followed by isopropanol, and spun dry. Labeled cDNA was resuspended in water and denatured on a 95°C block. Hybridization buffer (2×) was added to the probes and pipetted under a Lifterslip (Fisher, Pittsburgh, PA) covering the array. Arrays were hybridized in Corning (Lowell, MA) hybridization chambers overnight at 42°C in complete darkness. Arrays were washed in successively less stringent wash buffers, then spun-dry and stored in darkness until scanned on an Axon Instruments 4000B Scanner using GenePix Pro (Molecular Devices, Sunnyvale, CA) software.

### Microarray statistical analysis

The LIMMA/Bioconductor/R statistical package was used for statistical analysis of the intensity data from the arrays [79], [82], [83]. NORMEXP was used for background correction [84], followed by LOESS correction within arrays [85]. Specialty microarrays pose special problems during normalization; the LOESS normalization procedure has been found to be valid for arrays with as many as 20% of probes showing differential expression [86]. Detoxification genes make up approximately 20% of the genes spotted on the array, so LOESS normalization was used. DUPCOR from the LIMMA package was used to estimate the correlation between duplicate spots on the arrays [83]. Filtered corrected intensity values were fitted to a linear model and then ranked in order of evidence for differential expression using EBAYES. An intensity filter was applied to remove spots with average intensity values less than the intensity of negative control spots. A p-value<0.05, after false-discovery-rate correction [87], was established as the cutoff for genes differentially expressed.

### Gut dissection and measurement

The hindgut and midgut were dissected from 20 three-day-old bees fed on candy containing honey, pollen or propolis extracts, as were guts from bees fed unaugmented candy, by pulling on the sting with forceps. Dissected midguts were immediately separated on a chilled glass Petri dish and frozen in liquid nitrogen. Frozen guts were ground in a mortar using a pestle and RNA was extracted using Trizol. Parallel bioassays were set up to provide guts for morphological characterization; these midguts were dissected, stretched on a glass Petri dish by dragging with forceps, and measured across their widest girth to the nearest 0.1 mm with a dissecting microscope fitted with an ocular micrometer.

### Northern blot analysis

Expression of five CYP6AS subfamily gene transcripts, chosen based on their expression in the microarray experiments, was assayed with a northern blot. Probes specific for the entire P450 transcript were labeled with [α-32P]dATP (Amersham Biosciences) and purified using G-50 packed column. Total RNA (20 µg of each sample) was heated in loading buffer at 65°C for 15 min and separated on 1% formaldehyde-agarose gel by electrophoresis. After transferring RNA to Hybond-XL nylon membranes (Amersham Biosciences), the membranes were hybridized with probes following the manufacturers' procedures for these membranes. Northern blots were visualized using x-ray film exposed for 4 h, a duration chosen to minimize signal saturation.
